# Electrophysiological approaches to informing therapeutic interventions with deep brain stimulation

**DOI:** 10.1038/s41531-024-00847-3

**Published:** 2025-01-20

**Authors:** Atefeh Asadi, Alex I. Wiesman, Christoph Wiest, Sylvain Baillet, Huiling Tan, Muthuraman Muthuraman

**Affiliations:** 1 Neural Engineering with Signal Analytics and Artificial Intelligence, Department of Neurology, University Clinic Würzburg, Würzburg, Germany; 2 Department of Biomedical Physiology and Kinesiology, Simon Fraser University, Burnaby, BC Canada; 3 MRC Brain Network Dynamics Unit, Nuffield Department of Clinical Neurosciences, University of Oxford, Oxford, UK; 4 Montreal Neurological Institute, McGill University, Montreal, Canada; 5 Informatics for Medical Technology, Institute of Computer Science, University Augsburg, Augsburg, Germany

**Keywords:** Parkinson's disease, Parkinson's disease

## Abstract

Neuromodulation therapy comprises a range of non-destructive and adjustable methods for modulating neural activity using electrical stimulations, chemical agents, or mechanical interventions. Here, we discuss how electrophysiological brain recording and imaging at multiple scales, from cells to large-scale brain networks, contribute to defining the target location and stimulation parameters of neuromodulation, with an emphasis on deep brain stimulation (DBS).

## Electrophysiological brain mapping to enhance neuromodulation targeting

Please see *Section 4* for an overview of fundamental concepts in signal processing for electrophysiology.

### Non-invasive brain recordings

Electroencephalography (EEG) and magnetoencephalography (MEG) are non-invasive methods for recording neurophysiological activity at the sub-millisecond time scale. Their unique temporal resolution enables the direct measurement of brain rhythms and other complex features of brain activity in relation to behaviour and symptoms. With high-density scalp recordings, EEG and MEG have substantial source mapping capabilities, especially when the recordings are geometrically co-registered with the individual’s brain anatomy obtained from structural magnetic resonance imaging (MRI). A range of inter-regional functional connectivity measures can also be derived from EEG and MEG source maps, which temporal resolution enables functional explorations across a wide frequency spectrum (from below 1 Hz to 300 Hz and above)^1^. Combined, these benefits enable the definition of neurophysiological traits for a diversity of neurological conditions. Symmetrically, they can also specify neuromodulation therapies, both for targeting in terms of anatomical location and stimulation parameters. In the next two subsections, we summarize the respective assets of EEG and MEG, with a focus on applications to movement disorders, including Parkinson’s disease (PD), essential tremor (ET), and dystonia.

#### Electroencephalography (EEG) and Magnetoencephalography (MEG)

Electroencephalography (EEG) and magnetoencephalography (MEG) are widely used neuroimaging techniques for studying brain activity. While EEG captures electrical signals through scalp electrodes, MEG detects the magnetic fields generated by neural activity using external sensors. Both techniques provide high temporal resolution, making them essential for understanding neurophysiological processes in real time.

EEG measures the summation of electrochemical signals as they propagate between neurons. These signals are generated by the synchronized activity of large populations of pyramidal neurons aligned geometrically^2^. While EEG offers high temporal precision and is relatively low-cost and non-invasive^3,4^, its spatial resolution is limited to detecting signals on the scale of centimeters^5,6^. Despite this, EEG has become a fundamental tool in cognitive neuroscience research and clinical applications, particularly for studying movement disorders.

Conversely, MEG^7^ measures the magnetic fields induced by neural activity and offers greater spatial resolution than EEG due to minimal interference from scalp and skull tissues. MEG signals originate from the same neural assemblies that generate EEG signals, primarily cortical pyramidal cells^8^. However, MEG is also sensitive to subcortical structures such as the amygdala and brainstem^9–11^, as well as action potential volleys^12,13^. With potentially millimetre-scale spatial resolution^14^ and the ability to capture both oscillatory and aperiodic signals^15,16^, MEG is highly effective for studying brain activations across time and space.

EEG and MEG have both been employed to investigate the alterations in neurophysiological activity associated with movement disorders. For example, in PD, EEG has detected abnormal beta-band oscillations at rest^17^, which are normalized by deep brain stimulation (DBS), leading to improved motor symptoms^18,19^. MEG, with its superior spatial resolution, has further refined these findings by showing how alterations in beta oscillations affect movement planning in PD patients^20^. MEG has also demonstrated the involvement of oscillatory activity across multiple frequency bands, which are crucial for motor control and cognitive functions in PD^21,22^. These frequency-specific neural markers are critical for refining neuromodulation therapies, including transcranial alternating current stimulation (tACS) and transcranial direct current stimulation (tDCS), which show promise in modulating motor and cognitive functions in PD patients^23–28^.

Both EEG and MEG are valuable for studying inter-regional brain connectivity. EEG studies have revealed altered directional coherence between the cerebellum and premotor cortex in patients with ET and PD, suggesting that the normal flow of information between these regions is disrupted in movement disorders^1,29^. MEG, with its ability to detect cortical-subcortical interactions, has further demonstrated how these disruptions extend to deeper brain structures, implicating cortico-thalamo-cerebellar circuits in the pathophysiology of tremors^30^.

In the case of ET, both techniques have provided critical insights into the neurophysiological basis of tremor. EEG has detected event-related potentials in peri-motor cortical regions at latencies of 0.9–13.9 ms after DBS onset^31^, aligning closely with findings from invasive recordings^32^. Similarly, MEG has been used to trace rhythmic activity in the tremor frequency range, offering detailed spatial mapping of the neural oscillations underlying ET^33,34^. For dystonia, EEG and MEG have uncovered abnormal synchronizations within cortico-striato-pallido-thalamo-cortical circuits and cerebello-thalamo-cortical pathways^35,36^, highlighting the complex network dysfunctions driving dystonic movements.

Although EEG and MEG have traditionally been regarded as measuring cortical activity due to their sensitivity to large-scale synchronized neural activity near the brain’s surface, both techniques can detect deeper brain signals with the right experimental designs. MEG, in particular, has been shown to pick up signals from subcortical structures, such as the amygdala and brainstem, with greater signal-to-noise ratio when using advanced source localization strategies^30^. The integration of MEG with invasive electrophysiological methods has further linked subcortical dysfunctions to cortical activity patterns, deepening our understanding of brain networks involved in movement disorders^37,38^.

Together, EEG and MEG continue to provide complementary insights into the spatiotemporal dynamics of brain activity, and their combined use has proven instrumental in advancing both our theoretical understanding and therapeutic interventions for movement disorders like PD, ET, and dystonia.

### Invasive brain recordings

The spatial resolution of existing non-invasive recording methods is not sufficient to examine single neuron activity or local field potentials. Invasive recordings such as local field potentials (LFP) and micro-electrode recordings (MER) fill this gap and provide essential information about neural activity at smaller spatial scales in patients with movement disorders.

#### Local field potential (LFP)

Local field potentials (LFP) are the electrical potential in the extracellular space surrounding neurons and can be recorded in human participants using invasive electrodes. This is done most commonly in patients with movement disorders in the context of surgery, e.g., during implantation or battery replacement of DBS. These recordings measure the combined local neurophysiological activity of the implanted nucleus and surrounding tissues, and often capture oscillatory patterns relevant for treatment of movement disorders^39,40^.

The majority of DBS-LFP research has focused on the STN, as it is the most prominent DBS target for in treating motor symptoms in PD patients. LFP recordings were used to first demonstrate alterations of oscillatory activity in the basal ganglia of patients with PD^39,41^, which was once contentious but is now considered a pathophysiological hallmark of the disease. These alterations are normalized by dopamine therapy and related to the severity of motor impairments^40,42^. Further research using LFP recordings has demonstrated that the STN, in collaboration with the globus pallidus externus, serves as the originator of pathological somato-motor cortical beta activity in PD^43^. LFP recordings from the STN have also revealed neurophysiological alterations relevant to non-motor functions in PD, particularly involving lower frequencies ranging from 5 to 13 Hz^44^. Frequency-defined functional connectivity can also be assessed between deep-brain nuclei with LFP recordings. For instance, 3–10 Hz coherence between LFP recordings of the GPi and STN, the GPi and thalamus, and the thalamus and cortex are all related to the severity of parkinsonian tremor^45–47^.

LFP recordings have also revealed that heightened low-frequency (4–12 Hz) oscillatory activity of globus pallidus internus (GPi) neurons are a promising modifiable marker of dystonia^48^. The severity of dystonic symptoms correlates with the magnitude of these low-frequency GPi oscillations^49,50^, and they are suppressed by therapeutic DBS with proportional clinical improvement^50–53^. Consequently, the GPi has emerged as the main target for implanting DBS electrodes in dystonic patients.

#### Microelectrode recording (MER)

Microelectrode recording (MER) involves the insertion of a fine micro-electrode into brain tissue to record focal electrophysiology. This electrode measures the activity of multiple cells simultaneously and the resulting data need to be post-processed to sort between the contributions of individual neurons. While studies utilizing MER in patients with PD are limited, those that have been conducted primarily focus on localizing, identifying, and confirming target structures for neuromodulation. As the main target for DBS placement in PD patients, the STN is the focus of most MER studies. Utilizing MER enables the identification of more accurate electrode placement^54–57^. Sensorimotor regions and other sub-territories of the STN can also be demarcated during DBS surgery based on MER^55^, as can the borders of the GPi for DBS in patients with dystonia and PD^58^.

MER has also proven valuable in targeting the ventral intermediate nucleus (VIM) of the thalamus for DBS in patients with ET^59^. By providing real-time electrophysiological feedback, MER enhances the precision of electrode placement during surgery. This technique helps surgeons to differentiate the VIM from adjacent structures, which is crucial for optimizing tremor suppression while minimizing side effects. Studies have demonstrated that the use of MER improves clinical outcomes for DBS in ET patients^59,60^, allowing for more accurate targeting and potentially reducing post-operative complications.

### Multimodal brain recordings

Recording electrophysiology simultaneously using multiple of the aforementioned tools can provide benefits that the methods do not possess in isolation. Most notable among these are improved spatial accuracy (EEG-MEG) and the ability to associate whole-cortex neurophysiological activity to that of deep-brain nuclei in real time (M/EEG-LFP).

#### Combined EEG-MEG recording

Despite their identical temporal resolutions, the combination of simultaneously-collected MEG and EEG for source imaging consistently demonstrates superior spatial accuracy compared to either modality alone^61^. This enhanced localization stems from the distinct signals detectable with each modality, rather than from a mere increase in the total channel number. MEG’s capability for more accurate source imaging, owing to low magnetic interference from intervening tissues^7^, complements EEG’s sensitivity in detecting activity of deeper subcortical areas^7^^,^^61–63^. The activity of the thalamus and cerebellum can also be identified more robustly with combined EEG-MEG compared to when only EEG is used^63^. Therefore, the integration of MEG and EEG data proves crucial for conducting spatiotemporal studies of the whole human brain with maximal resolution^64^.

Despite this potential, relatively few studies have employed combined EEG-MEG to investigate the effects of movement disorders and DBS therapy. Future research should leverage these benefits towards the study of patients with movement disorders.

#### Combined EEG-LFP recording

Concurrent EEG and striatal LFP recordings have suggested that beta activity in the striatum is influenced in a task-dependent manner following dopamine depletion in PD^65–67^. These results indicate that the absence of dopamine in the sensorimotor striatum does not eliminate typical oscillatory patterns or introduce new frequencies of oscillations, and instead selectively reinforces inherent ones. This reinforcement primarily affects oscillations below 55 Hz, with the frequency band implicated dependent on the task being performed. Notably, this enhancement emerges only after extensive training.

Regarding the effects of STN-DBS on cortical activity, patients with PD exhibit peak frequency enhancement and power reduction in the alpha band^68^, and cortical alpha power has been positively correlated with clinical improvement following stimulation^69^. Suppression of beta oscillations in the temporal cortex^70^ and enhanced high gamma oscillatory activity in the right frontal cortex^70^ during DBS are also associated with motor improvement in PD.

EEG-LFP recordings have been employed to investigate connectivity between deep brain structures and the cortex in patients with movement disorders. For instance, in patients with PD and dystonia, movement-related coherence with the motor cortex is reduced to the STN and GPi, respectively^46,71,72^. This research has also distinguished between high (13–20 Hz) and low (20–30 Hz) beta oscillations in their relevance to PD symptoms^73–76^, with the effect of DBS on cortico-pallidal coherence in PD patients manifesting as a decrease in the high-beta range. Coherence analysis between cortical EEG and LFP signals in the GPi of patients with dystonia has identified two distinct types of dystonic symptoms^77^: alpha band power and coherence are associated with phasic symptoms, while delta power is linked to tonic symptoms.

#### Combined MEG-LFP recording

The most common combination of concurrent MEG with deep-brain neurophysiological recordings is in the context of deep brain stimulation (DBS) of the subthalamic nucleus (STN) to treat movement impairments in patients with PD. Despite its efficacy across a range of disorders^78^, the mechanisms underlying the multifaceted effects of DBS remain unclear^37,79^. MEG is particularly useful in the study of these mechanisms, as it is not as susceptible as EEG to the electromagnetic artifact caused by active DBS^37^ and to the changes in tissue electrical conductance resulting from the burr holes required for DBS electrode implantation^38^. Current artefact removal and attenuation techniques enable advanced analyses of MEG-DBS concurrent recording, including during active DBS stimulation^37,38^.

Brain network analyses have come at the forefront of current approaches to understanding DBS mechanisms^80^, with MEG contributing valuable insights^37^. Here too, the frequency-signature of these networks is considered as a key parameter of their clinical significance. For example, beta-frequency signalling between the STN and (pre-)motor regions are distinct from those between STN and temporal cortices in the alpha band^81,82^. Dysfunction of cortical beta-frequency networks is linked to motor impairments via deficient dopaminergic signalling from the substantia nigra (SN)^83^, indicating the potential role of STN-DBS in compensating for loss of SN integrity by restoring balanced levels of beta-network connectivity along the hyper-direct pathway. Indeed, although both local STN beta activity and STN-cortical beta coherence are reduced by the application of DBS^73,84^, the amount of beta-band coherence between STN and cortical regions better predicts DBS efficacy than local STN measures of spectral power^85^.

Non-invasive measures of cortico-STN beta coherence may help personalize neuromodulation parameters. Potentially, the timing, amplitude, and frequency parameters of DBS may be adapted for each individual to optimally reduce pathological beta synchrony while limiting secondary tissue scarring and desensitization. In a similar vein, brain-computer interfaces have been used to optimize DBS in patients with PD, but have only considered local STN beta synchrony measures to adjust DBS parameterss^86^. Further research combining DBS-LFP recordings with MEG is required to better understand the cognitive and affective impacts of DBS in patients with PD and other disorders. For example, recent work has shown that DBS applied during specific phases of decision making impacts the delay to decision^87^, an effect related to how DBS interacts with local STN beta oscillations. Using a similar paradigm during MEG would help understand how this effect may be mediated by network-level interactions across the entire cortex.

**Fig. 1:**
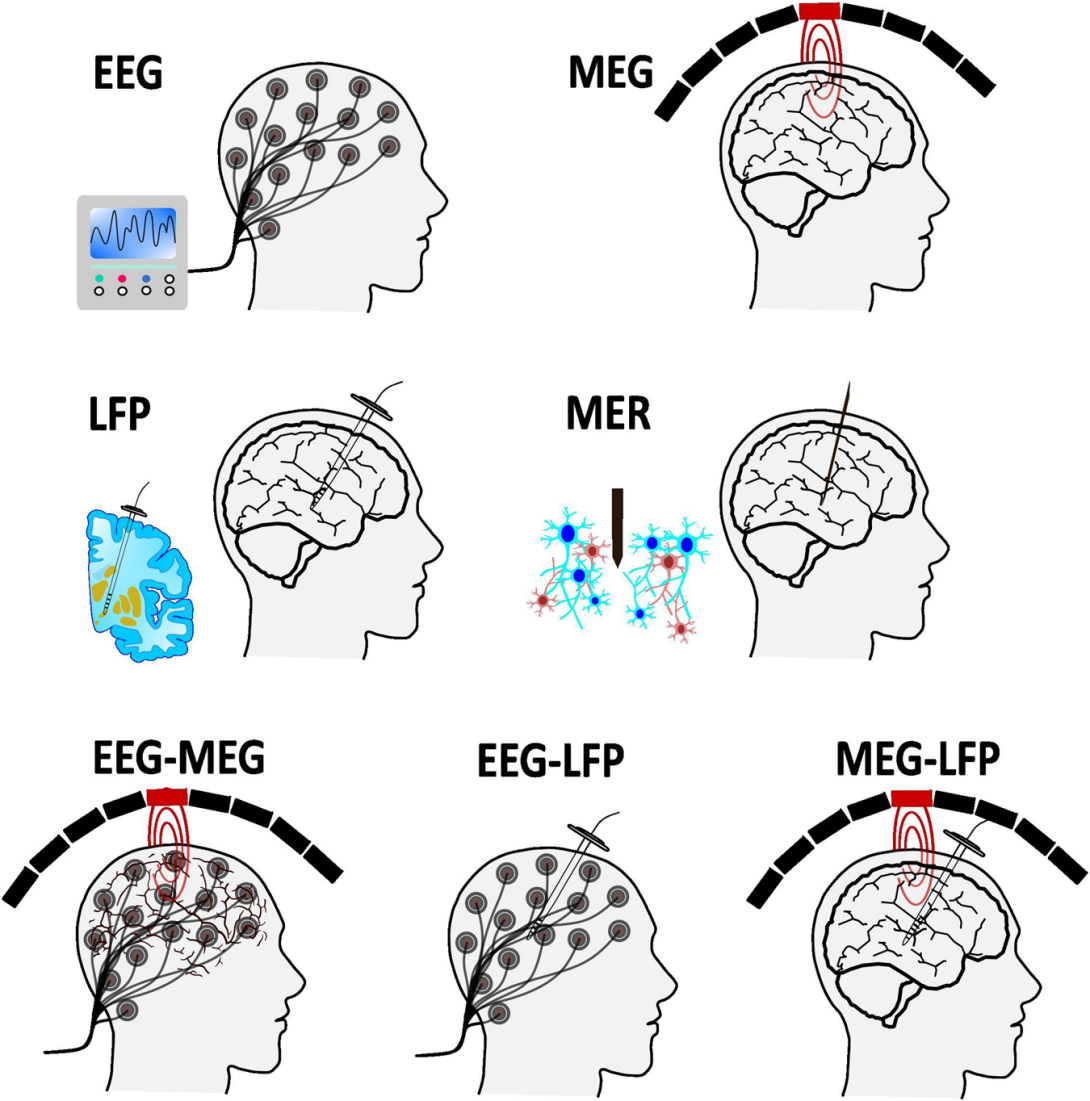
Schematic representation of single and combined electrophysiology methods.

**Fig. 2:**
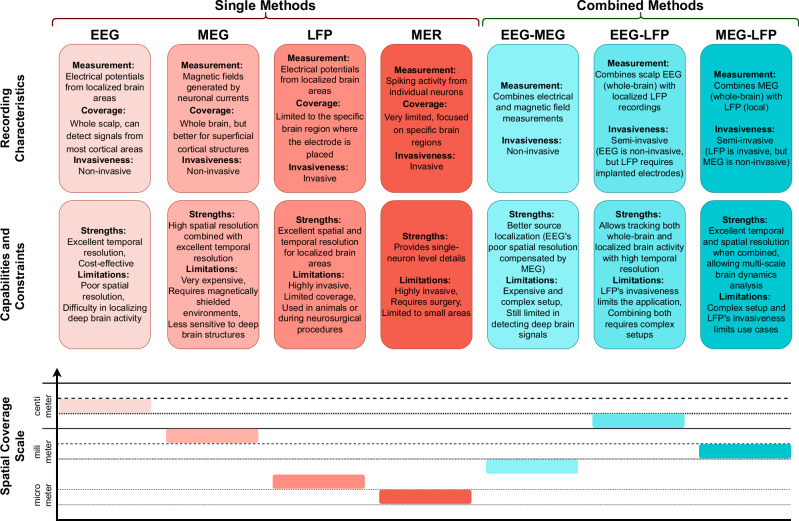
Characteristics and Comparative Analysis of Electrophysiological Recording Methods. This figure presents the strengths, limitations, and spatial resolution of the single and combined electrophysiological techniques discussed in the paper.

To summarize this section and provide a visual insight, a schematic representation of single and combinedelectrophysiological methods, along with a comparative analysis of electrophysiological recording methods, isdepicted in Figs. 1 and 2, respectively.

## DBS-induced spectral changes in the subthalamic LFP

One way of studying mechanisms underlying DBS and personalising stimulation is to investigate DBS-induced or evoked LFP changes in the subthalamic nucleus such as beta/gamma power suppression, evoked resonant neural activity (ERNA), finely-tuned gamma (FTG) oscillations and the aperiodic exponent of the power spectrum.

DBS-induced power suppression in the STN has been reported in multiple studies, with a focus on the beta range given the correlation with bradykinesia and rigidity in PD patients^19,84,88,89^. Notably, this power suppression is not specific to the beta range and extends into the low gamma range^5^. Beta and low gamma oscillations are almost instantaneously suppressed when stimulation is switched on and remain low throughout continuous DBS^90^. Once stimulation is switched off, both activities return to baseline more slowly, with low gamma recovering faster than beta activity^19^^,^^84^^,^^88–90^. DBS-induced power suppression has been suggested as an indicator of good lead placement and may help explain the mechanism underlying DBS: high-frequency stimulation desynchronises excessive beta activity which allows for relay of physiological activity through STN^91^. The link between beta activity and Parkinsonian symptoms, as well as its rapid suppression by classical DBS, has made beta activity a salient marker for aDBS. However, beta activity is limited by its sensitivity to noise contamination during movement. Furthermore, beta activity decreases at night^92,93^, which would result in an undesired proportional reduction of stimulation intensity and could be counterproductive in patients that profit from DBS during sleep^93–95^.

ERNA presents as a high-frequency, under-damped oscillation that can be observed in the STN and GPe/i of PD patients after DBS to STN or GPi^96,97^. ERNA is believed to arise from rhythmic inhibitory input from prototypic GPe neurons to STN^98,99^, however, simpler models utilising a single population of glutamatergic neurons can also model ERNA modulation during continuous STN-DBS by assuming synaptic failure as an underlying mechanism^100^. ERNA responses comprise fast and slow dynamics^90,98^: high-frequency DBS modulates its amplitudes and latencies over the first ten pulses, and skipping a single pulse affects subsequent ERNA pulse-responses^98^. Responses of ERNA dynamics to DBS reach a steady state after about 70 seconds^90^, and once DBS is stopped they slowly return to baseline levels on a timescale reminiscent of beta power recovery^98^.

Within STN, ERNA is highly focal and localises to a similar hot spot as the optimal one for clinical DBS benefit^101,102^. Several groups have also reported correlations between ERNA and clinical scores or beta activity^90^^,^^97^^,^^103–105^, and ERNA is modulated by dopaminergic medication^98^. Therefore, ERNA has been suggested as a marker for DBS contact selection and outcome prediction^106^. In addition, ERNA has been recorded during general anaesthesia which makes it a suitable intraoperative marker for lead placement^107^, and scales with increasing stimulation frequency and intensity^98^. ERNA can also be recorded from the STN of patients with cervical dystonia, which lacks the widespread neurodegeneration observed in PD^101^, suggesting that ERNA may be present in the healthy STN.

FTG is narrowband activity between 60–90 Hz that can be induced by levodopa or DBS, and less often can be recorded in the absence of either^108^. It is modulated by movement and both cortical FTG^108,109^ and cortical-subcortical FTG coherence^108,110^ have been suggested as potential markers for dyskinesia in PD patients. However, FTG is observed in only a subset of patients and is more prevalent in the superior margin of the STN, which may limit its clinical use^108^. In light of spurious horizontal line artefacts at sub-harmonics of stimulation, FTG entrainment has to be interpreted with care. DBS-induced subthalamic FTG is not locked at a sub-harmonic of stimulation and decreases with continuous DBS, this activity is unlikely to be artefactual^111^. Inversely, cortical FTG is entrained at a subharmonic of stimulation, but is affected by levodopa administration and disappears as DBS intensity is increased, which again makes this activity unlikely to be artefactual^112^.

Long considered noise, the aperiodic exponent of the power spectrum has now been shown to be modulated by several physiological changes and affected by excitation-inhibition balance in several studies of animal models and humans^113–115^. Aperiodic exponents of high-frequency (30–100 Hz) subthalamic LFPs increase with levodopa and high-frequency DBS, in keeping with increased STN inhibition effects^116^. As these exponents must be calculated over relatively long periods to reduce noise, they may only be useful in conjunction with slow beta-triggered aDBS^117^.

## Electrophysiological biomarkers which can foster closed-loop deep brain stimulation

In this section, we aim to elucidate the electrophysiological biomarkers that could facilitate the optimization of closed-loop adaptive DBS (aDBS). Classic DBS employs an open-loop stimulation approach, wherein electrical impulses are continuously delivered to the target tissue without any feedback mechanism. In contrast, emerging closed-loop approaches administer electrical stimulation based on the ongoing electrophysiological activities of the target (or connected) regions^110^. These cortical and/or subcortical electrophysiological feedback signals must be recorded and analysed in real-time alongside delivery of the stimulation pulses, with a specific emphasis on the control policies employed to adjust stimulation delivery^118^.

As mentioned above, PD is characterized by exaggerated beta oscillations in the LFP recordings of STN in patients, which are closely associated with motor symptoms^46,72^. The consistency and magnitude of these findings have positioned them as a potential biomarker for DBS feedback in PD^119,120^. The optimal DBS biomarker should vary meaningfully from patient to patient in a way that is representative of each individual’s symptom profile. Studies have revealed that beta oscillations can be divided into two functionally distinct sub-ranges: low-beta (13–20 Hz) and high-beta (20–30 Hz) oscillations, which seem to signal pathological and healthy motor function, respectively^73,121,122^. Consequently, it has been proposed to use low-beta frequency activity instead of canonical beta oscillatory power as a biomarker in aDBS^123^. A combination of several frequency ranges has been suggested to result in a more accurate detection of tremor in PD patients^124,125^. For instance, low-frequency (3–14 Hz) oscillatory activities in the basal ganglia have been proposed as a biomarker for tremor and non-motor symptom severity (e.g. impulsivity and depression) in PD patients^126,127^. Slowing of cortical neurophysiology across various frequency bands in PD has been linked to both cognitive and motor impairments. Moreover, this slowing has been shown to be sensitive to individual patient profiles and can be measured non-invasively, making it another emerging target for biomarker development^21,22^.

In dystonia, there is an enhancement of GPi activity from 4–12 Hz that is closely linked to the severity of symptoms^49,52^, making this frequency range a promising biomarker for aDBS in dystonic patients^53,128^. One study^50^ showed that in the dystonic and parkinsonian GPi, low-frequency and beta alterations, respectively, manifest as increases in phasic bursts characterized by episodes of relatively fast synchrony followed by intervals of quiescence. Further, the application of aDBS in the GPi was feasible and well-tolerated in both diseases. This indicates that heightened low-frequency burst amplitudes could serve as valuable feedback for guiding GPi-aDBS interventions in dystonia.

Unlike in PD and dystonia, robust neurophysiological biomarkers directly correlated with symptoms are lacking in ET, representing a challenge for aDBS in this patient group. Current candidate biomarkers instead include the assessment of tremor activity using external sensors such as accelerometers or electromyography (EMG)^128^, which measure inertial acceleration and muscular electrical impulses, respectively. Combined EEG-EMG recordings can be used to evaluate cortico-muscular interactions and show great promise in identifying biomarkers during clinical rehabilitation for neuromotor diseases^129^. Two primary approaches have been suggested towards this goal. Tremor amplitude may be utilized, where the onset of a tremor episode triggers stimulation based on predetermined amplitude thresholds, thus reducing tremor severity^130,131^. It has also been suggested consider tremor phase: stimulating the motor cortex out-of-phase to disrupt synchrony may modulate tremor severity^132,133^. Though both approaches have only been demonstrated through proof-of-concept studies, these results suggest that adaptive stimulation based on tremor amplitude and phase could be beneficial in reducing symptoms in ET. Further validation with chronic stimulation in real-world settings and direct comparisons with conventional stimulation therapies are essential.

## Fundamental concepts in signal processing for electrophysiology

Understanding signal processing concepts is critical for interpreting electrophysiological findings and their implications for therapeutic interventions. This section outlines key terms and principles related to signal processing that are essential for comprehending the methodologies employed in electrophysiology.

**Power bands**: Refers to specific frequency ranges of brain oscillations (e.g., delta: 2–4 Hz, theta: 5–7 Hz, alpha: 8–12 Hz, beta: 15–30 Hz, gamma: > 30 Hz). Each band is associated with different computational processes and cognitive and motor functions in the brain.

**Frequency resolution**: The ability to distinguish between different frequencies of brain activity in an electrophysiological recording. Higher resolution allows for more precise identification of activity in faster frequencies.

**Temporal resolution**: Refers to the precision of timing in capturing changes in neural activity and is directly related to frequency resolution. Different modalities (e.g., EEG, MEG) have varying temporal resolutions, affecting how quickly each can detect changes in brain activity.

**Signal-to-Noise Ratio (SNR)**: A measure that compares the level of a desired signal to the level of background noise. High SNR is essential for electrophysiological recordings, especially when trying to discern patterns of brain activity that are subtle or highly variable.

**Spatial resolution**: The ability to localize the origins of brain activity accurately. Different modalities offer different spatial resolutions, affecting the understanding of where in the brain specific electrophysiological changes occur.

**Filtering techniques**: Methods used to isolate specific frequency bands from raw “broadband” data, which can include low-pass, high-pass, band-pass, or notch filters.

**Event-related potentials/Fields (ERPs/ERFs)**: Measured electrophysiological responses in the brain that follow sensory, cognitive, or motor events. These are typically quantified after averaging across multiple stimulus presentations, and understanding their amplitude, latency, and other characteristics can help refine therapeutic interventions.

**Functional connectivity**: A class of analytical approaches that are used to assess the statistical interactions between electrophysiological signals from different brain areas. These measures are used to examine functional networks between regions, and how these networks may be influenced by disease states and therapeutic interventions.

**Spectral analysis**: Techniques such as the Fast Fourier Transform (FFT) that decompose electrophysiological time series into their constituent frequencies, allowing for the examination of different frequency bands.

**Aperiodic activity**: Non-oscillatory/arrhythmic signals that can provide insights into the underlying biophysical and computational features of an electrophysiological signal, such as the relative excitability of a brain region.

## Supplementary information

Supplementary Information for Review Paper: Key Concepts in Neurophysiological Data Analysis for Parkinson’s Disease

## Data Availability

All data analyzed during this study are included in this published article.
