# Supplementary material for: Electrophysiological approaches to informing therapeutic interventions with deep brain stimulation
Source: NPJ Parkinsons Dis. 2025 Jan 20;11:20. doi: 10.1038/s41531-024-00847-3 (PMC11747345; doi:10.1038/s41531-024-00847-3)
Supplement: Supplementary file 1 — Supplementary Information for Review Paper: Key Concepts in Neurophysiological Data Analysis for Parkinson’s Disease [file 41531_2024_847_MOESM1_ESM.docx]

**Supplementary Information for Review Paper: Key Concepts in Neurophysiological Data Analysis for Parkinson’s Disease**

**1. Power Bands**

Power bands are frequency ranges of brain oscillations crucial for understanding motor control and cognition. These frequency bands include:

Delta (1-4 Hz): Linked to deep sleep and motor control.

Theta (4-8 Hz): Associated with attention, learning, and memory.

Alpha (8-12 Hz): Reflects relaxed wakefulness and attention.

Beta (13-30 Hz): Strongly tied to motor control, commonly altered in movement disorders.

Gamma (30-100 Hz): Involved in higher cognitive functions and fine motor activity.

In PD: Abnormal beta-band activity (excessive synchrony) is often present and correlates with motor rigidity and bradykinesia.

In dystonia: Changes in alpha and beta bands have been observed, with reduced beta activity linked to motor symptoms. Dystonia often shows excessive low-frequency oscillations.

In essential tremor (ET): Increased theta and beta band activity is linked to tremor, while changes in gamma activity are sometimes observed during tremor episodes, reflecting alterations in motor networks.

**2. Frequency Resolution**

Frequency resolution refers to how precisely different frequencies in brain activity can be distinguished. It is essential for identifying abnormal oscillations in each movement disorder and defines as:
$\Delta f=\frac{band width}{spectral lines}$,

in which the band width is the maximum frequency that can be analyzed, and the spectral lines is referred to the total number of frequency domain data points.

In PD: High-frequency resolution helps in distinguishing pathological beta oscillations from normal brain rhythms.

In dystonia: Fine-tuned frequency resolution is needed to detect subtle shifts in low-frequency bands (such as the alpha or theta range) that may contribute to the disorder.

In ET: Distinguishing tremor-specific frequencies (typically 4-12 Hz) from normal activity is crucial, and higher frequency resolution aids in identifying tremor onset and its relation to other brain rhythms.

**3. Temporal Resolution**

Temporal resolution determines how closely the measured activity corresponds to the timing of the actual neuronal activity. Temporal resolution is critical for capturing rapid changes in brain activity:

In PD: High temporal resolution from techniques like EEG or MEG allows detection of real-time fluctuations in beta oscillations, especially during movement initiation or while at rest.

In dystonia: Temporal resolution is key to identifying the timing of abnormal muscle contractions and how they relate to brain oscillations, especially during involuntary movements.

In ET: Detecting tremor onset, frequency, and phase shifts in tremor-related oscillations relies on high temporal resolution, enabling the identification of when tremors start or are exacerbated.

**4. Signal-to-Noise Ratio (SNR)**

In terms of definition, SNR is the ratio of the desired signal (e.g., brain activity) to undesired background noise. In other words, SNR is the ratio of signal power to the noise power:

$SNR\left( dB \right)=\frac{P_{SIGNAL}}{p_{NOISE}}$,

P denotes the power and decibels (dB) is the unit of SNR expression.

Improving SNR is essential across all movement disorders.

In PD: High SNR is required to clearly distinguish pathological beta activity from other signals, especially during tremors or bradykinesia.

In dystonia: Given the often-erratic muscle contractions, obtaining a clear signal is challenging. High SNR ensures proper analysis of cortical signals driving abnormal postures.

In ET: Recording tremor-related activity requires a high SNR to differentiate between physiological tremor (normal oscillatory activity) and pathological tremor.

**5. Spatial Resolution**

Spatial resolution refers to the ability to precisely detect and distinguish electrical signals from different locations within neural tissues. High spatial resolution is crucial for accurately mapping the electrical activity of individual or small groups of neurons, allowing researchers to observe intricate patterns of neural communication. The resolution is determined by the electrode size, the number of electrodes in an array, and their proximity to the cells or tissue of interest. Fine spatial resolution enables more detailed and localized measurements, essential for understanding the functional architecture of the nervous systems.

In PD: Techniques like fMRI or deep brain stimulation (DBS) electrode recordings allow precise localization of abnormal activity in regions like the subthalamic nucleus or basal ganglia, which are deeply involved in the disease.

In dystonia: Spatially resolving abnormal activity in the cerebellum, thalamus, and cortex helps understand the disordered movement patterns and informs targets for interventions.

In ET: High spatial resolution is important to localize tremor-generating activity, often traced back to the cerebellum or thalamus, which are crucial nodes in the tremor network.

**6. Filtering Techniques**

In signal processing, filtering techniques such as low-pass, high-pass, and band-pass filters are essential for isolating, enhancing, or suppressing specific frequency components within a signal. Each filter type serves distinct purposes, particularly in analyzing data.

1. Low-Pass Filter (LPF)

Definition: A low-pass filter allows signals with frequencies below a specified cutoff frequency to pass through while attenuating frequencies above this threshold.

Applications: Commonly used to smooth data and remove high-frequency noise, such as electrical interference in EEG recordings.

2. High-Pass Filter (HPF)

Definition: A high-pass filter permits signals with frequencies above a specified cutoff frequency to pass while attenuating frequencies below this threshold.

Applications: Used to eliminate low-frequency noise, such as slow drifts or DC offsets, and to emphasize rapid signal changes.

Use in Neuroscience: HPFs are frequently applied to EEG data to remove slow fluctuations, allowing researchers to concentrate on higher-frequency brain activity.

3. Band-Pass Filter (BPF)

Definition: A band-pass filter allows signals within a specific frequency range (between a lower and upper cutoff frequency) to pass through, attenuating frequencies outside this range.

Applications: Widely utilized to isolate specific frequency components for analysis, such as particular brainwave bands in EEG studies.

**7. Event-Related Potentials (ERPs)**

Event-Related Potentials (ERPs) are time-locked electrical responses of the brain to specific sensory, cognitive, or motor events, measured using electroencephalography (EEG). ERPs reflect the brain's processing of stimuli and are represented as waveforms composed of various peaks and troughs, each associated with different cognitive processes such as perception, attention, memory, or decision-making.

**9. Spectral Analysis**

Spectral Analysis is a technique used in signal processing to study the frequency components of time-series data, particularly to understand how different frequencies contribute to a signal's overall structure. In various fields, such as neuroscience, physics, and engineering, it is employed to break down complex signals (e.g., EEG, sound waves, stock prices) into their underlying frequencies. By converting time-domain signals into the frequency domain, spectral analysis helps to identify periodicities, rhythms, or oscillations within the data, which are not easily observable in the raw signal.

**10. Aperiodic Activity**

Aperiodic activity refers to neural signals that do not exhibit regular or periodic patterns in their oscillations. Unlike rhythmic or periodic activities, which have well-defined frequencies and cycles (such as alpha or beta waves in EEG), aperiodic activity lacks a consistent temporal structure, making it more complex and variable.
